# Diversity and Evolution of Coral Fluorescent Proteins

**DOI:** 10.1371/journal.pone.0002680

**Published:** 2008-07-16

**Authors:** Naila O. Alieva, Karen A. Konzen, Steven F. Field, Ella A. Meleshkevitch, Marguerite E. Hunt, Victor Beltran-Ramirez, David J. Miller, Jörg Wiedenmann, Anya Salih, Mikhail V. Matz

**Affiliations:** 1 Section of Integrative Biology, University of Texas at Austin, Austin, Texas, United States of America; 2 Whitney Laboratory for Marine Bioscience, University of Florida, Saint Augustine, Florida, United States of America; 3 ARC Centre of Excellence in Coral Reef Studies, James Cook University, Townsville, Queensland, Australia; 4 National Oceanography Centre, University of Southampton, Southampton, United Kingdom; 5 Institute of General Zoology and Endocrinology, University of Ulm, Ulm, Germany; 6 School of Natural Sciences, University of Western Sydney, Penrith South DC, New South Wales, Australia; Cairo University, Egypt

## Abstract

GFP-like fluorescent proteins (FPs) are the key color determinants in reef-building corals (class Anthozoa, order Scleractinia) and are of considerable interest as potential genetically encoded fluorescent labels. Here we report 40 additional members of the GFP family from corals. There are three major paralogous lineages of coral FPs. One of them is retained in all sampled coral families and is responsible for the non-fluorescent purple-blue color, while each of the other two evolved a full complement of typical coral fluorescent colors (cyan, green, and red) and underwent sorting between coral groups. Among the newly cloned proteins are a “chromo-red” color type from *Echinopora forskaliana* (family Faviidae) and pink chromoprotein from *Stylophora pistillata* (Pocilloporidae), both evolving independently from the rest of coral chromoproteins. There are several cyan FPs that possess a novel kind of excitation spectrum indicating a neutral chromophore ground state, for which the residue E167 is responsible (numeration according to GFP from *A. victoria*). The chromoprotein from *Acropora millepora* is an unusual blue instead of purple, which is due to two mutations: S64C and S183T. We applied a novel probabilistic sampling approach to recreate the common ancestor of all coral FPs as well as the more derived common ancestor of three main fluorescent colors of the Faviina suborder. Both proteins were green such as found elsewhere outside class Anthozoa. Interestingly, a substantial fraction of the all-coral ancestral protein had a chromohore apparently locked in a non-fluorescent neutral state, which may reflect the transitional stage that enabled rapid color diversification early in the history of coral FPs. Our results highlight the extent of convergent or parallel evolution of the color diversity in corals, provide the foundation for experimental studies of evolutionary processes that led to color diversification, and enable a comparative analysis of structural determinants of different colors.

## Introduction

Fluorescent proteins (FPs) homologous to the green fluorescent protein (GFP) from the jellyfish *Aequorea victoria* are a fascinating protein family in many respects. Being only about 230 amino acid residues long, coral FPs, during their evolution, acquired an ability to synthesize several distinct types of fluorescent or colored moiety–the chromophore–from their own residues in two or three consecutive autocatalytic reactions, resulting in sometimes dramatically different spectroscopic characteristics [1]. Since the first description of Anthozoan members of the GFP family, these proteins have given rise to a variety of *in vivo* imaging techniques capitalizing on their unique spectral, physical or biochemical properties [2], [3], [4]. The ease with which coral FPs can be expressed and screened for phenotypic changes makes them ideal models for experimental studies in evolution of protein families, addressing in particular such important questions as convergent molecular evolution and the origins of molecular complexity [5], [6]. Last but not least, coral FPs are major determinants of the coral reef color diversity [7], [8], [9], [10], accounting for practically every visible coral color other than the brown of the photosynthetic pigments of algal symbionts (possible exception is the non-fluorescent yellow in some representatives of Poritidae and Dendrophylliidae that may be due to melanin-related pigments; C. Palmer, pers. comm.). A suggestion that the red appearance of some corals may be predominantly due to the phycoerythrins of cyanobacterial symbionts rather than intrinsic GFP-like proteins [11] was not supported in subsequent experiments [10]. FPs are the only known natural pigments in which the color is determined by the sequence of a single gene, which provides a unique opportunity to directly study the evolution of coral reef colorfulness at the molecular level [12].

Previous studies revealed four basic colors of coral FPs: three fluorescent ones (cyan, green, and red) and a non-fluorescent one (purple-blue) [9], [13]. Of these, only green and cyan share the same chromophore structure [14]. There are two types of red chromophore representing alternative ways to extend the “green” structure by means of an additional autocatalytic reaction. These chromophore types can be called DsRed-type [15] and Kaede-type [16] after the first proteins in which they were found. DsRed-like and Kaede-like chromphores are easily discernable by the shape of the excitation and emission spectra: Kaede-type proteins show much narrower major peaks with smaller Stokes shifts and a characteristic shoulder at 630 nm in the emission spectrum that makes them look remarkably like cyanobacterial phycoerythrins [11], [17]. In addition, there is a clear difference in the absorption spectrum of these types of red proteins under denaturing conditions. In 1M NaOH a DsRed chromophore is hydrolyzed resulting in a green-type chromophore structure with the characteristic absorption maximum at 445 nm [15]. In contrast, a Kaede-type chromophore in 1M NaOH absorbs with the maximum at 499 nm [10]. Kaede-type red proteins show a peculiar photo-induced color conversion: their final chromophore maturation stage that transforms the green-emitting GFP-like structure into the red chromophore requires violet light [16]. The photoconversion feature of Kaede-like red FPs made it possible to evaluate the half-life of these proteins *in vivo*, which turned out to be extremely long-on the order of 20 days [18]. Non-fluorescent purple-blue proteins (so-called chromoproteins or pocilloporins), which are characterized by high molar extinction coefficient but virtually no fluorescence [7], [19], possess yet another chromophore type, which is the isomerized version of the DsRed-like red chromophore [20]. There are three more derivatives of the DsRed-like structure, each observed once among FPs. The first one is the fragmented DsRed chromophore of the kindling fluorescent protein (KFP) from the sea anemone *Anemonia sulcata* [21], which was originally described as a chromoprotein [19]. Another one is a three-ring structure [22] found in the natural yellow fluorescent protein from *Zoanthus sp*. [23]. The third one is found in a mutant variant of DsRed called mOrange [24]. It is still largely unclear how changes in the FP’s amino acid sequence lead to such dramatic variations.

We have earlier reported the detailed analysis of remote homology relationships within the GFP superfamily [13], [25]. The aim of the present study was to systematically characterize fluorescent and/or colored GFP-like proteins found in reef-building corals (phylum Cnidaria, class Anthozoa, order Scleractinia), which represent the largest known repository of spectroscopic diversity of the GFP-like proteins. We describe 40 novel proteins that, along with the previously known ones, represent sampling from all six suborders of Scleractinia and cover 14 out of 21 families of corals. Our study provides an extensive knowledge base for biotechnological, evolutionary, and ecological studies utilizing GFP-like proteins as a subject or as a model.

## Results

### General characteristics of coral FPs

**Figure 1:**
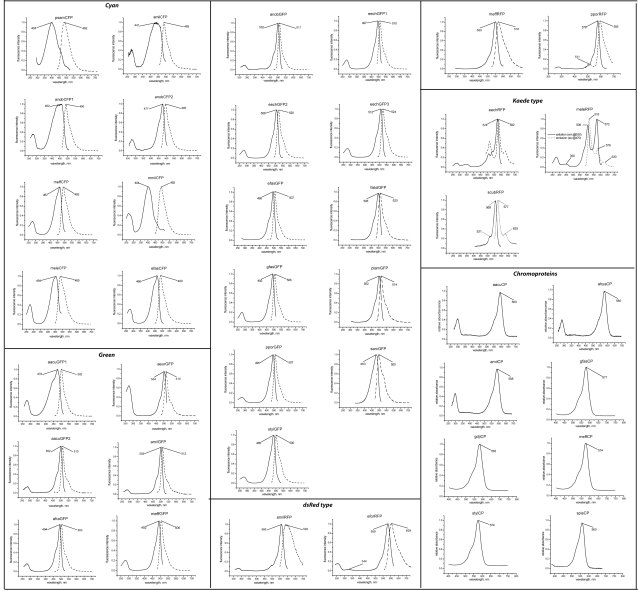
Excitation (solid lines) and emission (dashed lines) spectra of the newly cloned GFP-like proteins. Horizontal axis: wavelength in nanometers, vertical axis: relative fluorescence amplitude.

We used six sets of degenerate primers targeting the whole previously known diversity of Anthozoan FPs to isolate the coral FPs described here. Every coral species was subject to PCR trials with all degenerate primer combinations, resulting in identification of up to four distinct FPs from a single species. All but one of the newly cloned proteins fall into one of the four previously suggested color classes: fluorescent cyan, green, and red proteins, and non-fluorescent chromoproteins [5], [9]. A novel color type was represented by the protein from *Echinopora forskaliana* that exhibited the spectral phenotype intermediate between chromoproteins and DsRed-type red fluorescent proteins (hence its identifier is eforCP/RFP). Table 1 summarizes the spectral characteristics of the proteins cloned in this study, while Figure 1 shows all the excitation and emission spectra. According to the conservative method of semi-native gel electrophoresis [26], all the newly cloned FPs are tetramers or higher-order oligomers.

**Table 1:** Names, origins and spectroscopic characteristics of the newly cloned proteins.

		Host species	max	max	Quant.	Molar
	accession	*Genus species* (Sub-order, Family)	Excit	Emiss	yield	extinct.
CYAN						
amilCFP	AY646070	*Acropora millepora* (Archaeocoeniina, Acroporidae)	441	489	0.9	29500
anobCFP1	AY646072	*Acropora nobilis* (Archaeocoeniina, Acroporidae)	462	490	0.86	27600
anobCFP2	AY646071	*Acropora nobilis* (Archaeocoeniina, Acroporidae)	477	495	0.49	67200
meffCFP	DQ206381	*Montipora efflorescens* (Archaeocoeniina, Acroporidae)	467	492	0.55	88600
mmilCFP	DQ206392	*Montipora millepora* (Archaeocoeniina, Acroporidae)	404	492	0.9	42500
meleCFP	DQ206382	*Mycedium elephantotus* (Faviina, Pectiniidae)	454	485	0.74	47400
efasCFP	DQ206397	*Eusmilia fastigiata* (Meandriina, Meandrinidae)	466	490	0.77	40333
psamCFP	EU498721	*Psammocora sp.* (Fungiina, Siderastreidae)	404	492	0.96	30800
GREEN						
aacuGFP1	AY646069	*Acropora aculeus* (Archaeocoeniina, Acroporidae)	478	502	0.61	36900
aacuGFP2	AY646066	*Acropora aculeus* (Archaeocoeniina, Acroporidae)	502	513	0.71	93900
aeurGFP	EU498722	*Acropora eurostoma* (Archaeocoeniina, Acroporidae)	504	515	0.67	145700
afraGFP	AY647156	*Agaricia fragilis* (Fungiina,Agariciidae)	494	503	0.61	100800
amilGFP	AY646067	*Acropora millepora* (Archaeocoeniina, Acroporidae)	503	512	0.67	75200
anobGFP	AY646068	*Acropora nobilis* (Archaeocoeniina, Acroporidae)	502	511	0.6	96200
eechGFP1	DQ206383	*Echinophyllia echinata* (Faviina, Pectiniidae)	497	510	0.75	124200
eechGFP2	DQ206395	*Echinophyllia echinata* (Faviina, Pectiniidae)	506	520	0.69	109800
eechGFP3	DQ206396	*Echinophyllia echinata* (Faviina, Pectiniidae)	512	524	0.45	120600
efasGFP	DQ206385	*Eusmilia fastigiata* (Meandriina, Meandrinidae)	496	507	0.8	125800
fabdGFP	EU498723	*Favites abdita* (Faviina, Faviidae)	508	520	0.54	116800
gfasGFP	DQ206389	*Galaxea fascicularis* (Meandriina, Oculinidae)	492	506	0.73	102500
meffGFP	DQ206393	*Montipora efflorescens* (Archaeocoeniina, Acroporidae)	492	506	0.58	92000
plamGFP	EU498724	*Platygira lamellina* (Faviina, Faviidae)	502	514	0.96	98600
pporGFP	DQ206391	*Porites porites* (Poritiina, Poritidae)	495	507	0.54	98200
sarcGFP	EU498725	*Sarcophyton sp.*(Octocorallia, Alcyoniidae)	483	500	0.96	76700
stylGFP	DQ206390	*Stylocoeniella sp.* (Archaeocoeniina, Astrocoeniidae)	485	500	0.7	86700
DsRed-type RED						
amilRFP	AY646073	*Acropora millepora* (Archaeocoeniina, Acroporidae)	560	593	0.49	90900
meffRFP	DQ206379	*Montipora efflorescens* (Archaeocoeniina, Acroporidae)	560	576	0.56	99600
pporRFP	DQ206380	*Porites porites* (Poritiina, Poritidae)	578	595	0.54	94900
Kaede-type RED						
eechRFP	DQ206387	*Echinophyllia echinata* (Faviina, Pectiniidae)	574	582	0.43	12100
meleRFP	DQ206386	*Mycedium elephantotus* (Faviina, Pectiniidae)	573	579	0.85	45600
scubRFP	AY646064	*Scolymia cubensis* (Faviina, Mussidae)	570	578	0.6	66400
CHROMO-RED						
eforCP/RFP	EU498726	*Echinopora forskaliana* (Faviina, Pectiniidae)	589	609	0.16	111300
CHROMOPROTEINS						
aacuCP	AY646077	*Acropora aculeus* (Archaeocoeniina, Acroporidae)	580	NA	NA	109000
ahyaCP	AY646076	*Acropora hyacinthus* (Archaeocoeniina, Acroporidae)	580	NA	NA	132000
amilCP	AY646075	*Acropora millepora* (Archaeocoeniina, Acroporidae)	588	NA	NA	87600
gfasCP	DQ206394	*Galaxea fascicularis* (Meandriina, Oculinidae)	577	NA	NA	205200
gdjiCP	DQ206376	*Goniopora djiboutiensis* (Poritiina, Poritidae)	583	NA	NA	110300
meffCP	DQ206377	*Montipora efflorescens* (Archaeocoeniina, Acroporidae)	574	NA	NA	118300
stylCP	DQ206378	*Stylocoeniella sp.* (Archaeocoeniina, Astrocoeniidae)	574	NA	NA	96600
spisCP	DQ206398	*Stylophora pistillata* (Archaeocoeniina, Pocilloporidae)	560	NA	NA	61000

### Colors

#### Cyan

Although cyan proteins possess the same chromophore as greens [14], their evolution in corals by means of positive natural selection [12] warrants their recognition as a separate color class since it indicates that cyan fluorescence must have a dedicated, although yet unclear, role in corals’ physiology. Cyan proteins typically have an emission peak between 485–495 nm, although more blue-sifted variants can occasionally be found, down to 477 nm [27] . The considerable variation in exact position of the maxima is in a large part due to the poorly defined peaks in the spectral curves. Cyan proteins have notably wider excitation and emission curves than greens: the width of the curves at half-height is about 55 nm for cyans compared to about 35 nm for greens. Cyan proteins typically show the lowest molar extinction coefficient of all the colors (Table 1). Two cyan proteins reported here (psamCFP and mmilCFP) exhibit dramatically blue-shifted excitation maximum (404 nm), suggestive of a predominantly neutral ground-state chromophore, which, as we show below, is due to the presence of glutamic acid in position 167 (numeration according to GFP from *Aequorea victoria*). This spectral modification was previously unknown in cyan fluorescent proteins, either wild-type or artificially generated mutant variants.

#### Green

Green fluorescent color is the most common in corals and is the most conspicuous of all the fluorescent colors *in situ* [8]. We discriminate green proteins from the cyans by position of emission maximum (>500 nm), which usually correlates with the narrow half-width of the excitation and emission curves (see above). Occasionally there are borderline cases such as aacuGFP1, which has a narrow “green-like” emission peak at 502 nm but a rather “cyan-like” blue-shifted and wide excitation peak (Fig. 1). The position of excitation maxima in the newly cloned green proteins (around 478–512 nm) indicates the predominance of an anionic ground chromophore state, although a group of closely related proteins from *Acropora* species (aacuGFP1, aacuGFP2, amilGFP and anobGFP) display a minor, but noticeable absorption peak at about 395 nm corresponding to the neutral chromophore state.

#### Yellow

There are two wild-type yellow fluorescent proteins with emission maxima between 525 and 570 nm known at the moment: zoanYFP from a Zoanthidea representative (emission max 538 nm) and a hydromedusan protein phiYFP (emission max 535 nm). Despite the significant extent of our survey, not a single protein of this color has been cloned from corals, although yellow fluorescence with emission maximum exceeding 530 nm has been occasionally observed [17]. zoanYFP and phiYFP represent two different solutions to achieve yellow fluorescence. While phiYFP contains a GFP-like chromophore in a modified environment, zoanYFP possesses a unique three-ring chromophore structure that seems to be a result of deviation from the DsRed-type chromophore synthesis pathway. Interestingly, as it will be discussed below, such an explanation is corroborated by the phylogenetic position of zoanYFP.

#### Red

Corals possess either DsRed-type or Kaede-type red fluorescent proteins. We find Kaede-type proteins mostly associated with scleractinian corals of suborder Faviina. In addition, Kaede-type proteins are found in at least one representative of the order Corallimorpharia (carribean mushroom anemone *Ricordea florida*) and at least one representative of the family Nephtiidae of the order Alcyonaria, *Dendronephtya sp*. Red fluorescent proteins from all other organisms studied thus far, including other suborders of reef-building corals (Scleractinia), all sea anemones (Actiniaria) and two more Corallimorpharia representatives, possess the DsRed-like chromophore.

#### Purple-blue

The non-fluorescent chromoproteins (also called pocilloporins [7]) are characterized by intense absorption with a molar extinction coefficient commonly exceeding 100,000 and virtually no fluorescence. The chromophore in the chromoproteins is an isomerized non-coplanar version of the DsRed-like chromophore [20]. A mutation was previously identified that results in the chromophore isomerization in chromoproteins, which converts them into far-red fluorescent proteins with the emission maximum above 600 nm. Although the quantum yield in such mutants is typically low, they show sufficient brightness to be considered useful biotechnology markers since they retain a high “chromoprotein-like” molar extinction. Another useful feature of the chromoproteins is the “kindling” behavior [19], [28], which makes them prospective photoactivatable markers. All but one chromoprotein from the order Scleractinia identified previously, as well as in this study, are very similar in sequence and fall into the same phylogenetic group. The only significantly different chromoprotein is the novel pink spisCP from *Stylophora pistilata*. Its absorption maximum is at 560 nm (Fig. 1), which represents a blue shift by at least 14 nanometers in comparison to other known coral chromoproteins (hence pink rather than purple appearance). It should be noted, however, that some of the chromoproteins that arose independently in sea anemones (order Actiniaria) possess similarly blue-shifted absorption: for example, chromoproteins asCP562 [29] and cgCP [30] possess absorption maxima at 562 and 571 nm, respectively.

#### Chromo-red

In addition to the above color classes proposed by Labas et al (2002), in this study we identified a protein eforRFP/CP from *Echinopora forskaliana* possessing rather unusual spectroscopic characteristics. This protein has a molar extinction coefficient exceeding 100,000 M^−1^, which is more typical of chromoproteins than of red fluorescent proteins; however, it also shows the considerable (0.16) quantum yield of red fluorescence. The fluorescence peaks at 609 nm (Fig. 1), which is never seen in wild-type coral red fluorescent proteins (all of which emit below 600 nm), but is rather typical of fluorescent mutants of chromoroteins along with the relatively low quantum yield [19], [30]. Its alkaline and acid denaturation behavior suggests the presence of a DsRed-like chromophore (not shown). Since the spectroscopic characteristics of this protein most closely resemble an artificially generated fluorescent mutant of a chromoprotein [19], [30] rather than a wild-type DsRed-like fluorescent protein, and given its isolated phylogenetic position (see below), we believe that it is warranted to recognize this protein as a representative of its own new color class, “chromo-red fluorescent protein”.

### Phylogeny

**Figure 2:**
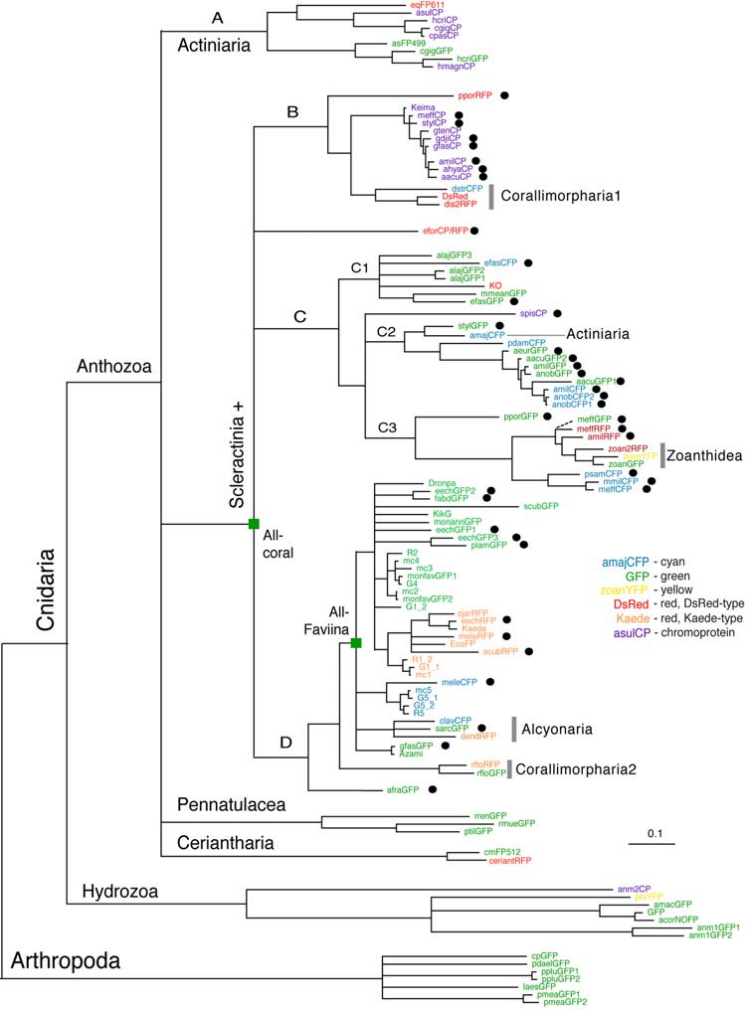
Bayesian phylogenetic tree of the cnidarian fluorescent proteins; Arthropoda FPs are shown as an outgroup. The edges with posterior probability less than 0.95 are collapsed. Black dots identify the proteins first described in this paper. Major clades and sub-clades are denoted, as well as the two reconstructed ancestral proteins (All-coral and All-Faviina). See legend for the color-coding of FP color classes. The position of meffGFP is tentative based on the short portion of its sequence that did not undergo gene conversion (see text for details). See Table S1 for the GenBank accession numbers corresponding to the protein names, and File S1 for the FASTA-formatted alignment of coding cDNA sequences.

The phylogenetic tree for Cnidarian fluorescent proteins is presented on Fig. 2. Scleractinian FPs form three separate clades, which we earlier designated B through D [13]. Each of these clades has a strong phylogenetic support (posterior probability essentially equals one), although the relationship between them remains unresolved. The grouping of these three clades to the exclusion of all but one FP from Actiniaria (clade A), Pennatulacea, and Ceriantharia is also very highly supported. Of the three Scleractinia clades, clade B is a clear example of a separate paralogous lineage that is retained in the genome, despite presence of multiple other FP genes, due to functional specialization. This clade contains non-fluorescent purple-blue chromoproteins from nearly every sampled family of Scleractinia (plus some from Corallimorpharia). Clades C and D underwent sorting among coral groups, so that we never find representatives of both within a single coral species. Each of these two clades contains a full complement of typical coral fluorescent colors: green, cyan, and red. Notably, the red fluorescent proteins of clade C are all DsRed-type, whereas they are Kaede-type in clade D. The specrtroscopically unique chromo-red protein eforCP/RFP from *Echinopora forskaliana* does not belong to any of the three major clades, although it constitutes their sister group. Some of the relationships between coral FPs resemble patterns suggested by the novel molecular-based coral phylogeny [31], [32]; however, its most basic subdivision into Robusta and Complexa is not recapitulated, most likely due to the extensive lineage sorting in the FP family.

#### Clade B

This clade is comprised mostly of the purple-blue non-fluorescent chromoproteins, which have been cloned from families Acroporidae, Pocilloporidae, Poritidae, Faviidae, Pectinidae, Oculinidae, and Dendrophyliidae. Most of these families yielded other FPs situated elsewhere within the tree. The tree on Fig. 2 includes only a small subset of the known chromoproteins, which we first describe in this paper. Omitting the others does not affect the overall phylogeny since all the chromoproteins of clade B are unusually similar in sequence, even the ones from different orders, Scleractinia and Corallimorpharia. Multiple, very similar chromoproteins can often be identified within a single species [33], suggesting a possibility of concerted evolution that may contribute to their sequence conservation. In addition to chromoproteins, clade B contains a group of corallimorpharian FPs, two of which are DsRed-type reds (including DsRed itself) and one cyan, plus a novel red FP from *Porites porites* (pporRFP) that occupies the most basal position within the clade and is also of the DsRed type. Thus far, clade B does not include any green FPs, which suggests that the common ancestor of this clade might have been either a red FP or a chromoprotein. Whether this is true or not, the grouping of all but one coral chromoproteins within one clade unequivocally indicates that the paralogous gene lineage responsible for the purple-blue color originated before the separation of scleractinian families.

#### Clade C

This clade received significant expansion through addition of the proteins reported here, as well as cloned by other laboratories since 2002. Ironically, clade C originally contained only the proteins from order Zoanthidea and the cyan protein from *Anemonia majano* (order Actiniaria), the placement of which within this clade we now tend to view as a phylogenetic complication (see Discussion). All of the other 24 proteins that joined clade C as a result of recent studies came from the order Scleractinia. Clade C includes three well-supported subclades (C1, C2 and C3, Fig. 2) each of which contains its own events of color diversification.

C1 subclade unites representatives from coral families Fungiidae (suborder Fungiina), Meandrinidae (Meandriina) and Rhizangiidae (Faviina), which may correspond to a grouping of these families into one of the Robusta subclades in the novel coral phylogeny [32]. C1 features diversification into cyan, green, and DsRed-type red fluorescent colors. At the divergence point of subclades C2 and C3 there is a surprise: the pink chromoprotein spisCP from *Stylophora pistillata* (suborder Archaeocoeniina, family Pocilloporidae). This protein clearly has evolved independently from the rest of coral chromoproteins. Interestingly, other representatives of the same coral family (but not of the same genus) yielded “conventional” chromoproteins of the clade B affiliation.

Subclade C2 contains green and cyan proteins from Archaeocoeniina suborder (families Acroporidae and Pocilloporidae) plus a cyan protein from sea anemone *Anemonia majano* (amajCFP, original name amFP486). Notable in this subclade are the multiple splits between cyan and green lineages: apparently these colors evolved from each other several times.

The C3 subclade is again a mixture of coral suborders: it contains a green protein from *Porites porites* (suborder Poritiina), cyan from *Psammocora sp.* (Fungiina) and one green and two red proteins from Acroporidae family (Archaeocoeniina); plus a group of proteins from order Zoanthidea. C3 is the most controversial subclade in the whole tree: its composition cannot be reconciled with any of currently considered phylogenies (see Discussion below). A notable feature of the subclade C3 is the secondary color radiation within Zoanthidea branch. The three Zoanthidea sequences correspond to red, yellow and green protein of which the red is basal; moreover, red fluorescent proteins amilRFP and meffRFP occupy the sequential basal positions with respect to the Zoanthidea branch. This renders it most likely that common ancestor of all the Zoanthidea proteins was a fluorescent red protein.

#### Clade D

Clade D includes several well-resolved nested subclades. The most basal branch corresponds to the green protein from coral genus *Agaricia* (suborder Fungiina). Moving up clade D, there is a group of FPs from order Corallimorpharia (mushroom anemones) and, rather unexpectedly, a group from order Alcyonaria (soft corals). The rest of clade D contains only FPs from families Faviidae, Mussidae, Trachyphyllidae, Oculinidae, and Pectinidae, all belonging to the suborder Faviina. With the exception of FPs from genus Galaxea (family Oculinidae), these proteins fall into three groups corresponding to cyan, green, and red fluorescent colors, of which cyan and red are monophyletic and green–paraphyletic.

### Gene conversion

Within the C3 subclade, there is an obvious case of gene conversion between green and red proteins of *Montipora efflorescens* (meffGFP and meffRFP): these two proteins are identical starting with the residue 66 (according to GFP numeration; in fact it is the chromophore-forming tyrosine) with not even a single third codon position substitution, whereas the N-terminal parts are substantially different (76% identity over 198 nucleotides of the corresponding coding region). The existence of such transcripts in the original *Montipora efflorescens* RNA sample was confirmed through independent RT-PCRs with gene-specific primers followed by sequencing of the product. Comparison to the closely related red fluorescent protein from *Acropora millepora* (amilRFP) revealed that amilRFP coding region is 90% identical to the 498 nucleotides of the converted meffRFP/meffGFP portion. Exactly the same level of identity is found between amilRFP and meffRFP within the remaining 198 nucleotides of the coding region, whereas the corresponding region in meffGFP is only 74% identical to amilRFP. This difference is highly significant (p<0.001) for the number of nucleotides involved. It can be concluded therefore that it was the portion of meffRFP gene that was copied into meffGFP via gene conversion and not the other way around. meffGFP was therefore excluded from the main phylogenetic analysis and its placement within clade C2 is tentatively based on the short unconverted portion of its coding sequence.

### Ancestral colors

For this study we reconstructed two ancestral proteins: one was the common ancestor of all coral proteins and the other an ancestor of all Faviina proteins (“all-coral” and “all-Faviina” respectively, Fig. 2). We applied a novel strategy of reconstruction to address the problem of uncertainty associated with the ancestral sequence prediction. Instead of synthesizing the protein having the most probable amino acid at each site, for each of the ancestral nodes we reconstructed five proteins in which the identity of the amino acid at a site was a result of random sampling from the underlying posterior distribution. Such a probabilistic mode of reconstruction has been proposed as a way to avoid bias towards higher stability and overall functional efficiency that could be expected in a consensus protein [34]. Using five samples, we expected to see the same phenotype in all the reconstructed variants, which would indicate that this phenotype represents a majority of all possible ancestral phenotypes with 95% confidence [35].

**Figure 3:**
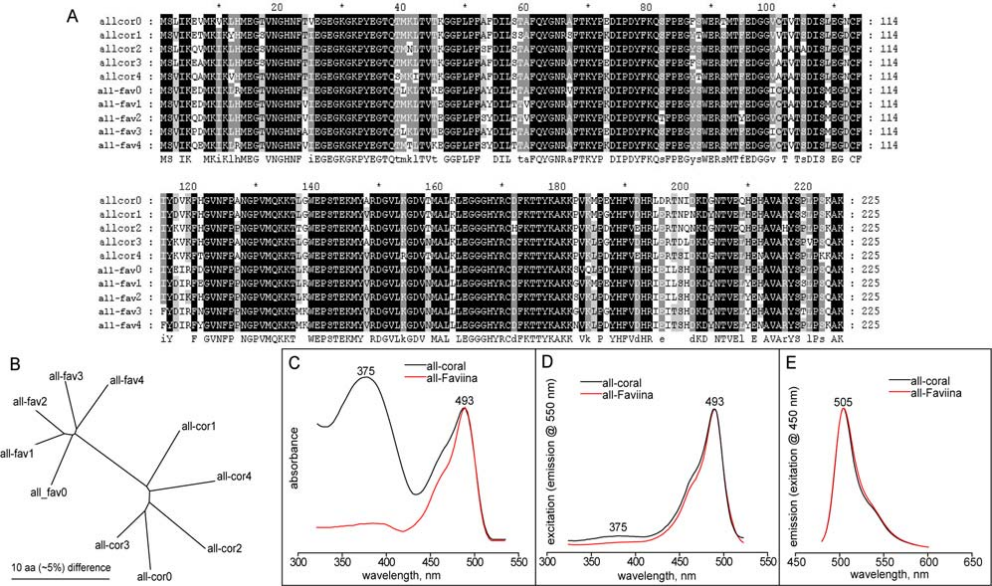
Analysis of ancestral proteins. A: alignment of the amino acid sequences of the reconstructed ancestral variants, five corresponding to All-coral ancestor (all-cor, 0 to 4) and five corresponding to All-Faviina ancestor (all-fav, 0 to 4). B: Unrooted neighbor-joining tree illustrating the degree of divergence between the synthesized ancestral sequences. C–E: absorbance, excitation, and emission spectra of a representative all-coral ancestor (black curves) and all-Faviina ancestor (red curves).

The sampled ancestral sequences corresponding to the all-coral ancestor differed between each other by 8–12%, all-Faviina sequences by 6–9% (Fig. 3 A, B). Despite these sequence differences, all the reconstructed variants exhibited practically identical fluorescence and absorbance phenotypes per ancestral node, with positions of the major peaks matching within 2 nm. This result indicates that the uncertainties of the ancestral sequence prediction did not affect the reconstructed ancestral phenotypes.

All of the reconstructed ancestral proteins demonstrated green emission (Fig. 3 E ) with the maximum of 505–506 nm and mirror-image excitation spectrum peaking at 493–495 nm (Fig. 3 D). Interestingly however, the absorbance spectrum differed rather dramatically between the all-coral and all-Faviina ancestors (Fig. 3 C): whereas all-Faviina absorbance spectrum was very similar to the excitation spectrum, suggesting the presence of typical GFP-like chromophore in its anionic ground state [36], [37], the absorbance spectrum of the all-coral ancestor featured a major peak at 375 nm that was practically not manifested in the excitation spectrum. This absorbance peak most likely corresponds to the chromophore in the neutral state, although it is more UV-shifted than in GFP from *A. victoria* (395 nm) or any of the cyan fluorescent proteins mentioned above (404 nm). Another distinctive feature that may actually be related to the UV-shift is that in the all-coral ancestor this chromophore state is very low-fluorescent (hence the almost complete absence of the 375 nm peak in the excitation spectrum, Fig. 3D), perhaps due to the lack of the proton transfer pathway that enables fluorescence after absorption in the neutral state [37]. The low molar extinction coefficient at 493 nm (31,000–33,000 M^−1^ for different variants) and low quantum yield (0.43–0.47) of the all-coral ancestor are ostensibly due to the large fraction of the protein being “locked” in the dark neutral state. The same parameters in the all-Faviina ancestor were on par with extant wild-type green proteins: its different sequence variants had molar extinction coefficient 88,000–100,000 M^−1^ and quantum yields of 0.67–0.80. All the reconstructed protein were tetrameric or higher order oligomeric according to the semi-native electrophoresis [26].

### Purple to blue shift in chromoproteins

**Figure 4:**
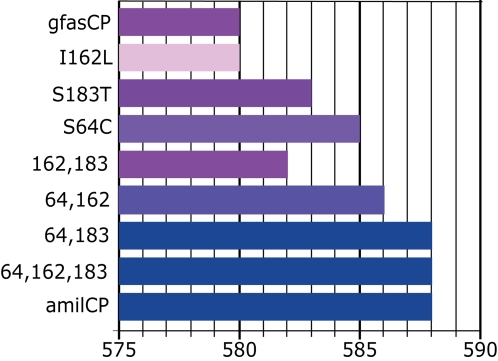
Positions of emission maxima in the mutated purple chromoprotein gfasCP in comparison to the blue amilCP. Horizontal axis is wavelength in nanometers, the bars indicate the position of the absorption peak in the mutant. The colors of the bars approximately correspond to the colors of the mutants. Mutations S64C and S183T were found to be responsible for the blue color in amilCP (numeration according to GFP). Mutation I162L results in a very slowly maturing protein, hence pale color of the corresponding bar. This effect is rescued by any of the other two mutations.

The chromoprotein amilCP is very similar to other coral chromoproteins in sequence; however, its absorption maximum (592 nm) is red-shifted by about 10 nm, making the protein appear blue instead of purple to the naked human eye. The closest homolog of amilCP is gfasCP, in comparison to which the amilCP protein has only four amino acid substitutions: S64C, I162L, S183T and S229P (numeration according to GFP from *A. victoria*). We investigated the effect of all combinations of these four mutations by introducing them into gfasCP and found that the blue phenotype was due to the substitutions at two sites: S64C and S183T (Fig. 4). The mutation at the fourth site (I162L) when introduced alone severely impaired the protein maturation: it took several days for soluble protein extract isolated after overnight induction of the expression in bacteria to develop color to the intensity comparable to what was seen already overnight in other variants. This effect was completely rescued by either of the two color-affecting mutations.

### Neutral versus anionic chromophore in cyan proteins

**Figure 5:**
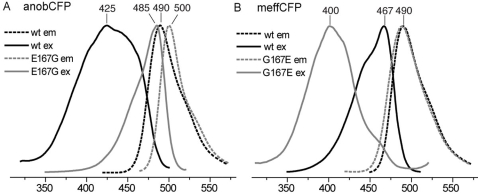
Glutamic acid at position 167 (numeration according to GFP) determines the “neutral-chromophore” phenotype in novel cyan fluorescenr proteins. A: Replacement of native E 167 by glycine in anobCFP leads to disappearance of 425 nm excitation peak and shift of both excitation and emission curves towards green. B: Reciprocal replacement G167E in a cyan protein meffCFP leads to the opposite results: virtually all the protein bulk started absorbing at 400 nm. Notably, the emission peak did not change.

Two novel cyan proteins from clade C, psamCFP and mmilCFP, feature an excitation spectrum very similar in shape to the wild-type GFP from *Aequorea victoria*, with the major peak at 404 nm (Fig. 1 and Table 1). It is very likely that such a spectrum, by analogy to GFP, indicates the predominantly neutral ground-state of the chromophore. In addition, in acroporid cyans (anobCFP and amilCFP) the excitation curve seems to contain a blue-shifted component, suggestive of a possible presence of the neutral chromophore in these proteins as well (Fig. 1 and 5). We noticed that in all these proteins, unlike all other FPs, the position 167 (GFP numeration) is occupied by glutamic acid. In a closely related cyan protein meffCFP, which does not have the 404 nm excitation band, position 167 is occupied by glycine. We mutated the residue 167 to glutamic acid in meffCFP and to glycine in anobCFP. In the former case the shortwave excitation band appeared and in the latter it vanished (Fig. 5), thus confirming the role of E167 in conferring the shortwave-excitation phenotype that is most likely associated with the neutral chromophore ground state.

## Discussion

### FP phylogeny versus host organism phylogeny

A substantial number of FPs from organisms not belonging to the order Scleractinia are intermingled within the three coral clades with high phylogenetic support. These include three other orders of hexacorals (sub-class Zoantharia), Corallimorpharia, Zoanthidea and Actiniaria–as well as, unexpectedly, order Alcyonacea (soft corals) from another sub-class (octocorals, Alcyonaria) (Fig. 2). Alcyonacea placement received additional support as a result of the present study in the form of yet one more protein , green sarcGFP, cloned from an Alcyonacea representative *Sarcophyton sp*. that groups together with the two previously known Alcyonacea FPs (clavCFP and dendRFP) within clade D. This is in strong contradiction with the current taxonomy that calls for the separation of subclasses (Alcyonaria and Zoantharia) preceding the separation of Zoantharia orders (Actiniaria, Zoanthidea, Corallimorpharia and Scleractinia). There are three ways to explain the FP/taxonomy incongruence: (i) spurious taxonomy; (ii) sorting of ancient paralogous gene lineages and (iii) horizontal gene transfer.

Unresolved taxonomic relationships between Scleractinia and Corallimorpharia may account for most of the discordance involving these two orders. Scleractinia have been proposed to originate several times from a Corallimorpharia-like ancestor by acquiring the ability to deposit a calcium carbonate skeleton [38]. More recent molecular analysis suggested a different scenario where Corallimorpharia originate once within Scleractinia by means of losing the skeleton [39]. Placement of Corallimorpharia proteins among Scleractinia is therefore expected. The polyphyletic origin of Scleractinia could also be responsible for the curious pattern of sorting of coral suborders between FP clades. On the basis of a combination of molecular and morphology characters at least two separate origins of Scleractinia have been proposed [31], [40]. These two groups of corals do not correspond to the traditional classification by suborders and have been named Complexa and Robusta referring to the prevailing mode of skeleton deposition [32]. There is some resemblance of this novel phylogeny in the FP tree, such as the C1 subclade uniting FPs from Fungiidae, Rhizangiidae and Meandrinidae (Robusta), close positioning (although not as sister groups) of Agariciidae and Oculinidae (Complexa) within clade D, as well as grouping of Poritiidae and Acroporidae (Complexa) within subclade C3. However, the FP phylogeny does not generally recapitulate the Complexa-Robusta split: FPs from groups that are thought to belong to Complexa (Poritiidae, Acroporidae, Agariciidae and Oculinidae) show no tendency to cluster into a unique clade (Fig. 2). For example, FPs from “robust” Pocilloporidae family (pdamCFP and spisCP) fall within subclade C2/C3 alongside the sequences from “complex” Acroporidae and Astrocoeniidae, seemingly in accord with the traditional taxonomic grouping of these families within Archaeocoeniina suborder.

We think that the best explanation for most of these discrepancies is paralogous lineage sorting. This explanation assumes that gene divergence within the ancestral genome preceded the organismal divergence. For example, to account for the occurrence of Alcyonaria proteins within clade D as well as deeper within the phylogeny (FPs from order Pennatulacea, Fig. 2) without the need to invoke pervasive polyphyly of Anthozoa orders, one may assume that the diversity of sequences bracketed by these two occurrences (i.e., all the major Zoantharia clades, from A to D) existed as paralogous genes within the genome of the common ancestor of Zoantharia and Alcyonaria [5]. The multiplicity of closely related genes accounting for each basic color in a closely investigated great star coral *Montastrea cavernosa* [9] suggests that the rate of gene duplication in the coral GFP-like gene family is indeed very high. The FP phylogeny may be predominantly reflecting the process of gene birth and death interspersed by selective sweeps leading to novel spectral features [12], which may considerably obscure the phylogenetic signal form the host organism evolution.

A group of sequences that does not quite fit any of the above explanations are Zoanthidea FPs, occupying a surprising position among scleractinian FPs within subclade C3 (Fig. 2). Unlike Corallimorpharia, order Zoanthidea was never suggested to have originated within Scleractinia by any analysis, so taxonomic uncertainty is not likely to be the case here. On the other hand, the position of Zoanthidea FPs within the FP tree is probably too derived to plausibly evoke the paralogous sorting explanation. In this case, it would require assuming a very unlikely scenario in which most of the FP diversity evolved as paralogous lineages in the common ancestor of Anthozoa orders and not much evolution happening since then. Zoanthidea FPs are not an artifact resulting from contamination by Scleractinian material, since the first Zoanthidea proteins were isolated before any coral material was searched for FPs, at least in our lab [23]. It is tempting to speculate that Zoanthidea acquired the FP gene from Scleractinia relatively recently via horizontal gene transfer, which may have been mediated by a common symbiont or pathogen. It is possible that some evidence of this event may be obtained through comparison of the genomic context of FP genes in Zoanthids and corals.

### Ancestral colors

Understanding the order and direction of the color transitions within the FP phylogeny is very important for studies of the structural determinants of color. To identify these, a typical comparative approach considers amino acid differences between the two most closely related proteins of different colors. However, in addition to the sites that are responsible for the color difference such a comparison will also reveal changes that were either neutral or related to a modification of other properties rather than color in both lineages since their separation. To narrow down the search, it is possible to compare the present-day proteins not to each other, but to their common ancestor. This at once removes half (on average) of the “ballast” mutations from consideration since only one of the two evolutionary lines of descent is considered. There is also an additional benefit of having the reconstructed ancestral proteins available for site-directed mutagenesis studies. Mutagenesis of present-day proteins can verify whether identities of certain residues are essential for the color; however, only changing these residues in the reconstructed ancestral protein in the evolutionary-forward direction can prove that such modifications are also sufficient [12]. We therefore reconstructed two ancestral proteins, all-coral ancestor and all-Faviina ancestor, which provide perspective to the history of coral color evolution.

We found that both ancestral proteins, the one at the root of the whole coral FP diversity as well as the much more derived protein ancestral to all Faviina FPs, were green and virtually identical in their excitation-emission properties (Fig. 3 D and E), although the all-coral ancestor had a peculiar absorbance spectrum indicative of the presence of the chromophore in a dark neutral state (Fig. 3 C). Such remarkable stability of ancestral fluorescence phenotype over considerable evolutionary distance is rather surprising, considering that in the present dataset a substantial number of non-green proteins appear very shortly after the diversification of the three major coral FP clades (B–D). These include the whole of clade B that does not have any green members, the chromo-red protein eforCP/RFP, the unusual pink chromoprotein spisCP that branches off early within clade C, as well as the red protein from Corallimorpharia that appears in the subclade that splits off in between the two reconstructed ancestral nodes (Fig. 2). It is reasonable to expect therefore that most of the coral FP tree has a “green trunk”, i.e., that nearly every ancestral protein that had green descendants was green. One likely exception from this rule may be Zoanthidea proteins, which conceivably evolved from a red fluorescent protein since they arise from within a group of red FPs within C3 subclade (Fig. 2). The evolution of green from red is achievable simply by inhibition of the third stage of autocatalysis during the red chromophore synthesis [15]. The appearance of the unique three-ring yellow chromophore in zoanYFP [22] also becomes less surprising if it is viewed as a result of deviation from the already complex pathway of the red chromophore formation. Given the diversity of chromophores in Zoanthidea FPs despite high sequence similarity, addressing this particular case of color diversification will be a promising subject for a future in-depth study.

The evolutionary significance of the strange absorption spectrum of the all-coral ancestor (Fig. 3C) is unclear at the moment, since none of its descendants show anything similar. It is tempting to speculate that this unusual phenotype reflects an important transitional stage that enabled quick diversification into a variety of colors early in the history of coral FPs. However, it is still possible that such an ancestral phenotype is, after all, a result of some unidentified systematic bias in the ancestral sequence prediction algorithm. Further ancestral reconstruction studies as well as in-depth structure-function analysis of the all-coral ancestral protein (beyond the scope of this paper) will clarify this issue.

It is important to add that the phenotype of the all-Faviina ancestral protein reported here was identical to the previously reconstructed version of the same node based on much less sequence data [6]. This indicates that our ancestral reconstruction results are robust to the inclusion of new sequences into the phylogeny.

### Structural determinants of color variation

The current dataset provides rich material for reconstruction of the evolutionary paths resulting in novel spectral features and identification of the structural determinants of color variation. In this paper, we addressed two cases of color change. Two mutations turned out to be responsible for the unusual blue color in chromoprotein amilCP: S64C and S183T (Fig. 4). Residue 64 is immediately adjacent to the chromophore-forming triad, while the 183th side chain is involved in the interface between monomers within a tetrameric FP structure. Interestingly, position 64 is also occupied by cysteine in an artificially generated far-red emitting mutant of DsRed, mPlum [41]. There is unexpected epistatic interaction of these two mutations with the third one, I162L, which dramatically slows down the maturation of the chromoprotein if introduced alone, but does not have such an effect in combination with either S64C or S183T. Interestingly, the mutation I162L makes the protein slightly bluer if combined with S64C. From this it is reasonable to speculate that if the blue color was indeed the target of selection, the natural order of mutations most likely was S64C, I162L, S183T, resulting in a gradual transition towards the blue color.

Two blue chromoproteins from sea anemones (order Actiniaria): aeCP (absorption maximum 597 nm) [42] and the remarkable cjBlue (absorption maximum 610 nm) [43] must be mentioned here. All the Actiniaria chromoproteins belong to the Actiniaria-specific clade A, and thus clearly arose independently from coral chromoproteins. Similar to amilCP, both aeCP and cjBlue contain C64 and T183-but so do many other Actiniaria chromoproteins that are purple. It can be speculated that, although the structural determinants of blue color in aeCP and cjBlue may include the same residues that we identified in amilCP, the non-fluorescent color variation in Actiniaria is due to some other mutations that also contribute to the blue color.

The second key spectrum-modifying mutation that we determined is the glutamic acid in position 167, conferring a novel excitation property to cyan proteins presumably indicative of a neutral chromophore ground state (Fig. 5). Such a modification was previously unknown in cyan FPs, either wild-type or artificially generated mutant variants, although the residue at position 167 has been previously implicated in contributing to the cyan phenotype in general [12], [44]. Neutral-chromophore cyan proteins, similar to GFP, may become valuable photoactivated markers [45] due to the proton transfer process characteristic of their photocycle [37].

### Understanding the function of coral FPs

Despite the great interest in discovering new FPs and adopting them for biotechnology needs, the progress in understanding their biological function (or functions) in non-bioluminescent organisms such as corals has been frustratingly slow. Currently there are several hypotheses based on indirect evidence, of which several or none may eventually turn out to be true. The ideas related to symbiosis with dinoflagellate algae of the genus Symbiodinium (zooxanthellae) include photoprotection (suggested by Kawaguti [46], [47] and substantiated by physiology data by Salih and co-authors [48]), fine symbiosis regulation [12], aposematic coloration, and masking the presence of algal pigments within coral tissues from herbivorous fishes [8]. Alternative explanations include deactivation of reactive oxygen species [49] and proton pumping [50], [51]. It should be noted that both of these latter hypotheses have been suggested based on the experiments with the original jellyfish-derived GFP, which has a neutral ground state chromophore and shows a peculiar proton transfer during the photocycle [37]. Until now neutral chromophores were not observed in coral FPs; however, this study reveals multiple such cases in cyan proteins. It is possible therefore that the proton-transfer photocycle, perhaps associated with either proton pumping or reactive oxygen species deactivation, constitutes part of the function of the cyan color in particular. Our recent statistical phylogenetic analysis of FPs from Faviina, coupled with the site-directed mutagenesis study, revealed that the new non-green colors (cyan and red) evolved under the pressure of positive natural selection, which means that the diverse colors must serve some essential function [12]. Multiple events of parallel evolution of the same colors highlighted by this present work strongly corroborate this result. We also found previously that a subset of residues arranged as an intra-molecular interface in Faviina FPs evolved under diversifying positive selection, suggestive of a “co-evolutionary arms race” with an unknown binding partner [12]. Although we chose to interpret these observations in light of the symbiosis-related functionality, other explanations may be equally probable, involving functions unrelated to symbiosis, and perhaps even not related to fluorescence or any light modification (such as deactivation of oxygen radicals) if different colors translate into different reactive properties. To finally settle the question of the function of coral fluorescence a series of studies is necessary, dedicated specifically to finding the ecological correlates of coral fluorescence variation. Spatial and temporal patterns of protein and gene expression have to be analyzed, as well as the tissue distribution of individual color types. Preferably, such a study should be conducted across color morphs of a single coral species for which the full complement of FP colors has been cloned. The present work suggests a promising model for such kind of research: *Acropora millepora*, which yielded all four principal colors (cyan, green, red and non-fluorescent blue) and is an emerging genomic model [52], [53]. Studies of genomic loci of coral GFP-like proteins may shed additional light on their evolutionary history, by generally improving the resolution of the phylogenetic tree and highlighting major transition events related to gene duplication and subfunctionalization. Such information will be invaluable for reconstructing the ancestral sequences and backtracking the phenotypic shifts, to get to the basics of color determination at the sequence level. Finally, very important for understanding the biological function of the coral GFPs will be to investigate their protein-protein interactions *in vivo*, which is especially interesting in relation to the putative molecular interface that is under positive natural selection [12].

## Materials and Methods

### Collection of samples

Samples (100–500 mg of tissue) of the Caribbean coral species (*Agaricia fragilis, Scolymia cubensis, Eusmilia fastigiata, Montastrea cavernosa,* and *Porites porites*) were obtained from the Florida Keys Marine Sanctuary under National Marine Sanctuary authorization FKNMS-2000-009. Samples of Indo-Pacific corals (*Acropora aculeus, Acropora millepora, Acropora nobilis, Acropora eurostoma, Echinophyllia echinata, Echinopora forskaliana, Galaxea fascicularis, Goniopora djiboutiensis, Montipora efflorescens, Montipora millepora, Mycedium elephantotus, Psammocora sp., Stylocoeniella sp.,* and *Stylophora pistillata)* as well as soft coral *Sarcophyton sp* were collected at several locations on the Great Barrier Reef, Australia under Marine Parks Permit #G05/13283.1.

### Cloning and expression of coral FPs

Total RNA was isolated from the organism using RNAqueous kit (Ambion) and amplified cDNA was prepared from it using SMART protocol [54]. The complete cDNA coding sequences for GFP-like proteins were obtain by modified Step-Out RACE [55], [56] using degenerate primers for homology cloning. For 5’ stage of RACE, 12 pairs of upstream-directed degenerate primers were used:[Table pone-0002680-t002]

**Table pone-0002680-t002:** 

cladeD_fav_1	ATHWTYTCDGTGGATGSYTCCCAYTT
cladeD_fav_n	TYMGGRAACRWCTGCTTGAAATA
cladeD_rest_1	ATCTCAATGCRRTGGTCCACAAA
cladeD_rest_n	TASTTTTRAAGTCACAYMGGTAAT
cladeD_agar_1	AATGCGGTGGTCTATGAAGTGA
cladeD_agar_n	TGCTTGAAAWAGTCTGGTATGT
cladeB_1	TAACMGGTCCATYRGRAGGAAAGTT
cladeB_n	AAATGGCARAGGTCCNCCCTTGGT
cladeC_rest_1	TTACRGGTCCATCAGCRGGAAAGTT
cladeC_rest_n	WWGABAGWATGTCMAAGGAGAAT
cladeC_zoanred_1	TTCTTAGCATCGCTGCGGTCTT
cladeC_zoanred_n	ACCATCCTTCAGAAGGAGGTACAT
cladeC_zoan_1A	TTTCATCACAGGTCCATCAGCAGGAAA
cladeC_zoan_nA	ACAYGARTTCTTGAAAWAGTCAAC
cladeC_zoan_1B	TTGCTATCACTGGTCCATTGTCAGGAAA
cladeC_zoan_nB	AAAAGATGGCTTGAAAWAGTCAAC
cladeC_styl_1	CATYAYGGGTCCRTYAGCRGGAAAGTT
cladeC_styl_n	AGWATGTCAAAGGARAATGSAAGG
cladeB2_1	GTCTTCYTYTGCAYAACRGGTCCAT
cladeB2_n	ACCRTCTTCRAAGKKCATGRHCCKYTCCCAT
cladeD2_1	WTCTCAGTGGATGGYTCCCA
cladeD2_n	RYRAAHRCYCTGTTKCCRTA
cladeC2_fung_n	CRGGRAAGTTVACSCCRACAAATT
cladeC2_fung_1	CCRCCTCCYDCVAGCTTKAGGWACAT

The “1” primers were used in the first 5’ RACE PCR reaction together with 5prox (5’ proximal adaptor primer [56]) for 12 cDNA samples from different organisms. The PCR reaction was performed in 96-well plate. The cycling parameters were: 94°C 40″- 55°C 30″-72°C 1′, 30 cycles. The product of the first PCR was used in the nested PCR reaction with “n” primers and Udist (universal adaptor primer, [56]). The cycling parameters: 94°C 40″- 55°C 30″-72°C 1′, 18 cycles.

The RACE products were cloned into pGEM-T vector (Promega) and sequenced (6–8 clones per each product). The sequences were assembled in SeqMan II software (Lasergene) and the contigs were checked for homology using BLASTX [57]. A pair of 3’-RACE primers were designed for each FP-related contig to amplify a complete open reading frame (ORF). The nested 3’-RACE primer corresponded to the very beginning of the ORF and contained a “translation initiation heel” [58]. After identification and sequencing of fluorescent clones, the inserts from them were re-amplified using the same 3’-RACE nested primer and a primer corresponding to the C-terminus of the ORF with six histidine codons inserted in front of the termination codon, as we described earlier [9]. The product of this amplification was cloned into pGEM-T vector and used to produce a heterologously expressed protein that could be purified by metal-affinity chromatography using Ni-NTA agarose (Qiagen).

### Spectroscopy

The fluorescent properties of isolated proteins were determined using LS-50B spectrofluorometer (Perkin Elmer Instruments); emission spectra were corrected for the dependence of photomultiplier sensitivity on the wavelength. Molar extinction coefficients of native proteins were determined from the absorption of the chromophore in denaturing conditions (in 1M NaOH) assuming a molar extinction coefficient of 44,000 for cyan, green, chromoproteins and DsRed-like red proteins[25] and 28,000 for Kaede-like proteins [10]. Quantum yields were determined by using either fluorescein (QY = 0.97) or sulforhodamine 101 (QY = 0.90) as a reference standard.

### Phylogenetic analysis

The coding DNA sequence alignment of the fluorescent proteins was assembled following the protein sequence alignment, that was in turn constructed by appending the previously reported alignment [25]. The appropriate model of evolution was identified as GTR+G+I [59] with the help of Modeltest software [60]. The phylogenetic analysis was performed using MrBayes 3.1 [61]. The MCMCMC chain was run for 1,500,000 iterations with a sample frequency of 200 resulting in 7,500 trees, of which the first 6,000 were discarded while summarizing the data. The analysis was run three times to ensure convergence.

### Probabilistic ancestral reconstruction

To reconstruct ancestral proteins sequences for the common ancestor of all coral proteins (“all-coral ancestor”) and the ancestor of all proteins from representatives of the Faviina suborder (“all-Faviina ancestor”) we used MrBayes 3.1 with the fixed amino acid-based model JTT [62]. Five sequences per ancestral gene were probabilistically assembled by drawing the site states from the underlying posterior distribution of state probabilities and synthesized individually as described earlier [6].

## Supporting Information

Table S1 List of GenBank accession numbers for all the sequences used in the phylogenetic analysis. The newly cloned sequences are shown in blue.
(0.04 MB PDF)

File S1 FASTA-formatted alignment of the coding sequences used for the phylogenetic analysis.
(0.10 MB DOC)
