# Supplementary material for: Diversity and Evolution of Coral Fluorescent Proteins
Source: PLoS One. 2008 Jul 16;3(7):e2680. doi: 10.1371/journal.pone.0002680 (PMC2481297; doi:10.1371/journal.pone.0002680)
Supplement: Table S1 — List of GenBank accession numbers for all the sequences used in the phylogenetic analysis. The newly cloned sequences are shown in blue. (0.04 MB PDF) [file pone.0002680.s001.pdf]

Cyan

amajCFP	AF168421
amilCFP	AY646070
anobCFP1	AY646072
anobCFP2	AY646071
clavCFP	AF168424
dstrCFP	AF168420
efasCFP	DQ206397
G5_1	AY182022
G5_2	AY182023
meffCFP	DQ206381
mmilCFP	DQ206392
meleCFP	DQ206382
mc5	AY181556
pdamCFP	AY679113
psamCFP	EU498721
R5	AY182017

Green

aacuGFP1	AY646069
aacuGFP2	AY646066
acorNOFP	AY151052
aeurGFP	EU498722
afraGFP	AY647156
alajGFP1	AY508123
alajGFP2	AY508124
alajGFP3	AY508125
amacGFP	AF435432
amilGFP	AY646067
anm1GFP1	AY485334
anm1GFP2	AY485335
anobGFP	AY646068
asFP499	AF545827
cmFP512	AF545830
cgigGFP	AY037776
cpGFP	AB185173
Dronpa	AB180726
eechGFP1	DQ206383
eechGFP2	DQ206395
eechGFP3	DQ206396
efasGFP	DQ206385
fabdGFP	EU498723
KikG	AB193294
G1_2	AY182020
G4	AY182021
Azami	AB107915
gfasGFP	DQ206389
GFP	P42212
hcriGFP	AF420592
laesGFP	AY268073
mc2	AY181553
mc3	AY181554
mc4	AY181555
meffGFP	DQ206393
mmeanGFP	AY155344
monannGFP	AY037766
monfavGFP1	AY679112
monfavGFP2	AF401282

Green (continued)

plamGFP	EU498724
pdaelGFP	AY268076
pmeaGFP1	AY268074
pmeaGFP2	AY268075
ppluGFP1	AY268071
ppluGFP2	AY268072
pporGFP	DQ206391
ptilGFP	AY015995
rfloGFP	AY037772
rmueGFP	AY015996
R2	AY182014
rrenGFP	AF372525
sarcGFP	EU498725
scubGFP	AY037767
stylGFP	DQ206390
zoanGFP	AF168422

DsRed type

amilRFP	AY646073
ceriantRFP	AY296063
dis2RFP	AF272711
eqFP611	AY130757
KO	AB128820
meffRFP	DQ206379
pporRFP	DQ206380
DsRed	AF168419
zoan2RFP	AY059642

Kaede type

cjarRFP	EF186664
dendRFP	AF420591
eechRFP	DQ206387
G1_1	AY182019
EosFP	AY765217
Kaede	AB085641
mc1	AY181552
meleRFP	DQ206386
R1_2	AY182013
rfloRFP	AY037773
scubRFP	AY646064

Yellow

phiYFP	AY485333
zoanYFP	AF168423

Chromo-Red

eforCP/RFP	EU498726

Chromo

aacuCP	AY646077
ahyaCP	AY646076
anm2CP	AY485336
amilCP	AY646075
asulCP	EF587182
cgigCP	AF363775
cpasCP	AF383155
gfasCP	DQ206394

Chromo (continued)

gdjiCP	DQ206376
gtenCP	AF383156
hcriCP	AF363776
hmagnCP	AY461714
Keima	AB209967
meffCP	DQ206377
stylCP	DQ206378
spisCP	DQ206398
